# Non-invasive brain stimulation as a tool to study cerebellar-M1 interactions in humans

**DOI:** 10.1186/s40673-016-0057-z

**Published:** 2016-11-16

**Authors:** Sara Tremblay, Duncan Austin, Ricci Hannah, John C. Rothwell

**Affiliations:** Sobell Department of Motor Neuroscience and Movement Disorders, UCL Institute of Neurology, London, WC1N 3BG UK

**Keywords:** Cerebellum, Non-invasive brain stimulation, Paired-associative stimulation, Primary motor cortex, Theta burst stimulation, Transcranial direct current stimulation, Transcranial magnetic stimulation

## Abstract

The recent development of non-invasive brain stimulation techniques such as transcranial magnetic stimulation (TMS) has allowed the non-invasive assessment of cerebellar function in humans. Early studies showed that cerebellar activity, as reflected in the excitability of the dentate-thalamo-cortical pathway, can be assessed with paired stimulation of the cerebellum and the primary motor cortex (M1) (cerebellar inhibition of motor cortex, CBI). Following this, many attempts have been made, using techniques such as repetitive TMS and transcranial electrical stimulation (TES), to modulate the activity of the cerebellum and the dentate-thalamo-cortical output, and measure their impact on M1 activity. The present article reviews literature concerned with the impact of non-invasive stimulation of cerebellum on M1 measures of excitability and “plasticity” in both healthy and clinical populations. The main conclusion from the 27 reviewed articles is that the effects of cerebellar “plasticity” protocols on M1 activity are generally inconsistent. Nevertheless, two measurements showed relatively reproducible effects in healthy individuals: reduced response of M1 to sensorimotor “plasticity” (paired-associative stimulation, PAS) and reduced CBI following repetitive TMS and TES. We discuss current challenges, such as the low power of reviewed studies, variability in stimulation parameters employed and lack of understanding of physiological mechanisms underlying CBI.

## Background

The cerebellum plays a fundamental role in the production and control of skilled movements [1, 2] via its outputs to both cortical and brainstem structures. Here we consider the evidence that it is possible to stimulate and influence the excitability of the cerebellum non-invasively through the scalp in conscious volunteers.

The main evidence that transcranial stimulation can activate neurones in the cerebellum comes from the work of Ugawa and colleagues who studied the specific connection between cerebellum and primary motor cortex (M1). Classically this pathway is comprised of the disynaptic dentate-thalamo-cortical (DTC) connection [3, 4] which exerts a facilitatory effect on the motor cortex. It originates from the dorsal region of the dentate nucleus and receives inhibitory input from likely targets of transcranial stimulation, the Purkinje cells in lobules VII and VIII of cerebellar cortex [2, 5]. Ugawa et al. showed that stimuli delivered by either high intensity electrical pulses applied across the mastoid processes or transcranial magnetic pulses around the inion reduced the excitability of corticospinal outputs from the M1 contralateral to the site of cerebellar stimulation if tested 5–6 ms later [6, 7]. This was termed cerebellar inhibition of motor cortex (CBI). They postulated that stimulation activated Purkinje cells which then inhibited ongoing excitatory output from dentate nucleus and removed facilitation from M1. The delay of 5–6 ms before suppression could be detected at M1 and was considered to be compatible with the estimated time for conduction and synaptic delays. This conclusion was supported by later findings showing that the effect was suppressed in patients with pathology affecting the cerebellar cortex or cerebellar output pathway [8]. It was also consistent with the finding that deep brain stimulation of the ventrolateral thalamus in patients with essential tremor could modulate CBI [9]. In addition to effects on corticospinal excitability, stimulation of cerebellum was also found to interact with other local circuits in M1 that were involved in short interval intracortical inhibition (SICI), long interval intracortical inhibition (LICI) and intracortical facilitation (ICF) [10].

These early experiments also highlighted a number of other factors that could overlap with this effect and confound the simple interpretation that all the effects were caused by stimulation of cerebellum. Because the surface of the cerebellum is some distance from the scalp, relatively strong stimuli have to be applied to suppress M1. This activates sensory afferents in the neck which themselves can suppress M1 excitability. Luckily the latency of this effect occurs later (7–8 ms), meaning that a relatively pure cerebellar effect can only be guaranteed by testing with cerebellum-M1 intervals of 5–6 ms [11]. A second consequence of the high stimulus intensities is that the stimulation can spread deeper into the brainstem and activate the corticospinal tract at the pyramidal decussation. This can be avoided by carefully finding the threshold for corticospinal activation and then reducing the intensity below this by 10 % [6]. Given the potential for activation of corticospinal fibres, it remains an open question as to whether there could also be activation of sensory afferents in the medial lemniscus. This would lead to a short latency suppression of M1 excitability analogous to short latency afferent inhibition (SAI) usually evoked by direct stimulation of peripheral nerve.

A final unknown concerns the idea that CBI is due to withdrawal of ongoing facilitation. We know that facilitatory effects can have a rapid onset, which is consistent with the known duration of the rising phase of a cortical (extrastriate and thalamocortical) excitatory post-synaptic potentials (EPSP, 1–2 ms: [12–14]). There are no comparable ways to estimate how rapidly removal of ongoing facilitation could take effect. If we imagine instantaneous halting of all ongoing EPSPs, then the time taken for activity to fall should equal the total duration of the last set of EPSPs that arrived, which is at least 5–7 ms [13]. This is much slower than the very rapid onset of CBI (1–2 ms). The situation is unclear and needs to be resolved. Nevertheless, given these caveats, cerebellar inhibition of M1 is a useful tool for testing connectivity in the dentato-thalamo-cortical pathway.

More recently, a number of other methods have been introduced in an attempt to produce long lasting, “plasticity-inducing” changes in cerebellar function. These employ repetitive transcranial magnetic stimulation (rTMS) and transcranial direct current stimulation (TDCS). The rationale is that when these are applied directly to M1, they change the excitability of corticospinal output for the following 30–60 min by mechanisms that involve early stages of synaptic plasticity in cortical neurones. The assumption is that similar effects might be seen over cerebellum since animal studies have shown that cerebellar Purkinje cells exhibit unique features of synaptic plasticity, involving both long-term depression and long-term potentiation [15].

The aim of this article is to review relevant literature concerned with the impact of cerebellar “plasticity” protocols on M1 measures of excitability and plasticity in both healthy and clinical populations. Results will be discussed with regards to the specific aspect of M1 neurophysiology that was assessed following cerebellar stimulation in healthy individuals. This will be followed by a short summary of the impact of cerebellar stimulation in clinical populations.

When reviewing the evidence, we have borne in mind the evolution of the much larger body of work in which the same or similar methods were applied to M1. In this case, early descriptions in small cohorts of volunteers appeared to be consistent with simple rules such as “high frequencies of rTMS increase and low frequencies decrease M1 excitability”, or “anodal TDCS excites whereas cathodal suppresses M1 excitability”. Later work, however, in larger populations has shown that the methods are highly variable, often with only 50 % of people responding in the “expected” way. The reasons for this are complex and probably multifactorial. Nevertheless, they probably explain a number of puzzles such as some of the failures to reproduce results and apparent contradictions in the literature. They might also be a factor that limits therapeutic potential.

### Review

A systematic review of the literature was performed using the following databases: PubMed (2000 to Mar 2016) and Medline (2000 to Mar 2016). The following search keywords were selected: “TDCS”, “transcranial direct current stimulation”, “theta burst stimulation”, “TBS”, “repetitive transcranial magnetic stimulation”, “rTMS”, “primary motor cortex”, “cerebellum”. Initially, 70 articles corresponded to our search criteria. After carefully reviewing the abstracts we identified 23 articles that specifically investigated the effects of cerebellar stimulation on primary motor cortex neurophysiology (hand muscles) in clinical populations and healthy individuals. We excluded studies that assessed the effect of cerebellar stimulation using only behavioural measures or imaging methods other than TMS. Subsequently, we read the full texts of the final sample and searched references for additional articles, which led to the inclusion of five additional papers. Studies were only included if they were published in English and described thoroughly their methodology. Our final sample comprised 28 publications.

### Primary motor cortex changes following cerebellar stimulation in healthy individuals

Three different type of plasticity protocols have been applied to the cerebellum: low and high frequency rTMS; intermittent and continuous theta burst stimulation (iTBS, cTBS); and TDCS or transcranial alternating current stimulation (TACS). The effects of these protocols when applied over M1 are considered to be well established, although they exhibit wide inter-individual variability (see [16–18] for methodological reviews). For instance, low frequency rTMS (≤1Hz) and cTBS are known to reduce M1 excitability presumably via modification of synaptic plasticity similar to long term depression, while high frequency rTMS (5–20 Hz) and iTBS are associated to increases in M1 excitability via long term potentiation-like mechanisms. TDCS is thought to induce similar bidirectional modifications of cortical excitability, i.e. decrease with cathodal stimulation and increase with anodal stimulation, presumably via changes in resting membrane potentials. Transcranial alternating current stimulation (TACS) can increase neuronal excitability through entrainment of desired neuronal firing frequency. When applied over the cerebellum, studies have generally employed the same stimulation parameters (e.g. duration, intensity) as for plasticity protocols over M1. A separate group of plasticity paradigms involves cerebellar-M1 paired-associative stimulation (CB-M1 PAS) [19]. This paradigm is thought to induce spike-timing dependent plasticity (STDP), by repeatedly pairing (120 pairs at a frequency of 0.25 Hz) a cerebellar afferent input with M1 suprathreshold TMS at different intervals (2, 6 and 10 ms).

The effects of these forms of cerebellar stimulation have been assessed on a range of outcome measures involving M1. Table 1 provides a description of each protocol. These include: 1) *corticospinal excitability* measured in terms of resting motor threshold (RMT), motor evoked potential (MEP) amplitude to standard suprathreshold TMS pulse and MEP recruitment curve (MEP_RC_); 2) *intracortical excitability* measures such as SICI ([20, 21]), LICI [22], cortical silent period (CSP: [23]), ICF [20], short interval intracortical facilitation (SICF: [24]), SAI [25] and long latency afferent inhibition (LAI [26]); and 3) *M1 plasticity* assessed via PAS [27, 28] and TBS.

**Table 1:** Description of TMS protocols assessing M1 activity

Measures	Protocol	Outcome
RMT	Smallest intensity of the SMO required to elicit MEPS of ≥ 50 μV	Synaptic excitability in M1 Excitability of axons in M1 activated by TMS
MEP	Average amplitude of MEPs using a fixed SMO (1 mV intensity, or percentage of RMT) or multiple intensities (recruitment curve: e.g. 100 to 150 % of RMT)	Global corticospinal excitability
CBI	Dual-coil: suprathreshold CS to the cerebellar cortex 5–7 ms before a suprathreshold TS over the contralateral M1	Excitability of the DTC pathway
SICI	Paired-pulse: subthreshold CS 2–3 ms before a suprathreshold TS over M1	Short duration GABAa-ergic inhibition
LICI	Paired-pulse: suprathreshold CS 100–200 ms before a suprathreshold TS over M1	Long duration GABAb-ergic inhibition
CSP	Suprathreshold TS applied during slight tonic contraction of target muscle	Long duration GABAb-ergic inhibition
SICF	Paired-pulse: suprathreshold CS 1.1–1.5, 2.3–2.9 and 4.1–4.4 ms before a subthreshold TS over M1	Excitability of cortical interneurons and I-waves generation
ICF	Paired-pulse: subthreshold CS 7–20 ms before a suprathreshold TS over M1	Cortical net facilitation involving glutamate
SAI	Pairing of a median nerve electrical stimulation 20–25 ms before a suprathreshold TS over M1	Sensory afferent inhibition mediated by Ach and GABAa-ergic inhibition
LAI	Pairing of a median nerve electrical stimulation 200 ms before a TS over M1	Sensory afferent inhibition (pathway unknown)

*ACh* acetylcholine, *CBI* cerebellar brain inhibition, *CS *conditioning stimulus, *CSP* cortical silent period, *DTC* dentate-thalamo-cortical pathway, *GABA* gamma-aminobutyric acid, *RMT* resting motor threshold, *LAI* long latency afferent inhibition, *LICI* long interval intracortical inhibition, *MEP* motor evoked potential, *SAI* short latency afferent inhibition, *SICI* short interval intracortical inhibition, *SICF* short interval intracortical facilitation, *ICF* intracortical facilitation, *SMO* stimulator output intensity, *TS* test stimulus

None of the types of cerebellar stimulation have been applied at an intensity sufficient to activate directly the dentate-thalamo-cortical connection. Thus any effects on M1 seem unlikely to be due to repeated application of CBI. They are more likely to involve persisting local changes in the cerebellum itself. A comprehensive description of the methodology and results is shown in Table 2 (rTMS and TBS), Table 3 (TDCS and TACS) and Table 4 (CB-M1 PAS). Table 5 gives a complete description of results for each outcome measure.

**Table 2:** Effect of cerebellar rTMS and TBS on primary motor cortex excitability

Authors	Sample size	Stimulation target(s)	Protocol	Parameters	Sessions	Target muscle	Coil size	Timing of measurements	Findings
Gerschlager et al. (2002) [29]	8 HC	Right CRB	1 Hz rTMS	500 pulses 40 % MSO Biphasic	1	Right and left FDI	CRB: double-cone (110 mm) M1: figure-of-eight (90 mm)	Pre/Post N1 (0, 5, 10, 15 min), Post N2 (20, 25, 30 min)	CRB and control target: ↑ MEP only in Left FDI
	5 HC	Right posterior neck (control)		As above	1	As above	Neck: figure-of-eight (90 mm)	As above	
Oliveri et al. (2005) [30]	10 HC	Left CRB (main experiment)	1 Hz rTMS	600 pulses 90 % RMT	1	Left FDI	M1 and CRB: figure-of-eight (70 mm)	Pre (3 blocks)/Post 0, 5, 10 min	↑ MEP, ICF ↔ SICI, CSP
	6 HC	Left CRB (time course)	As above	As above	1	As above	As above	Pre (3 blocks)/Post 0, 30, 60 min	↑ ICF (0–30 min)
	6 HC	Left CRB (ipsilateral hand)	As above	As above	1	Right FDI	As above	Pre/Post 0 min	↔ ICF
Fierro et al (2007) [31]	8 HC	Right lateral CRB (main experiment)	1 Hz rTMS	900 pulses 90 % RMT (inion)	1	Right FDI	M1 and CRB: figure-of-eight (70 mm)	Pre (2 blocks)/Post 0, 10 min	↔ SICI, MEP ↓ ICF
	4 HC	Right posterior neck (control)	As above	As above	1	As above	As above	As above	↔ MEP, SICI, ICF
	4 HC	Right lateral CRB (time course)	As above	As above	1	Right APB	As above	Pre/Post 5, 10, 15, 20 min	↑ MEP (15–20 min)
	8 HC	Right lateral CRB (time course)	As above	As above	1	Right FDI	As above	Pre/Post 0, 10, 20 min	↓ ICF (0–20 min)
Langguth et al. (2008) [48]	10 HC	Medial CRB Right lateral CRB	1 Hz rTMS	1000 pulses 120 % RMT	4 randomized crossover	Right ADM	M1 and CRB: figure-of-eight (70 mm)	Pre/Post 0	1 Hz: ↑ SICI, ICF, ↔ RMT
		Medial CRB Right lateral CRB	10 Hz rTMS						10 Hz: ↔ SICI, ICF, RMT
Koch et al. (2008) [34]	10 HC	Left lateral CRB	cTBS	600 pulses 80 % AMT	20 subjects randomly assigned to 7 exp.	Left and Right FDI	M1 and CRB: figure-of-eight (90 mm)	Pre/Post 0, 15, 30, 60 min	↓ MEP, SICI
	12 HC	As above	As above	As above		As above	As above	Pre/Post	↑ LICI; ↔ SICF
	6 HC	Left cervical root (control)	As above	As above		Right FDI	As above	As above	↔ MEP, SICI, LICI
	6 HC	Left lateral CRB	As above	600 pulses 90 % AMT		As above	As above	As above	↓ MEP, SICI; ↑ LICI
	10 HC	As above	iTBS	600 pulses 80 % AMT		As above	As above	Pre/Post 0, 15, 30, 60 min	↑ MEP, LICI; ↓ICF
	10 HC	As above	As above	As above		As above	As above	Pre/Post	↓ LICI, ↔ SICF
Koch et al. (2009) [48]	10 PD with LID	Left lateral CRB	cTBS	600 pulses 80 % AMT	2 pseudo-randomized	Left FDI	CRB: figure-of-eight (70 mm)	Pre/Post	Active (vs sham): ↓ SICI; ↑ LICI
			Sham	600 pulses 40 % AMT					
Popa et al. (2010) [32]	10 HC	Right CRB	1 Hz rTMS	900 pulses 90 % Adj.RMT	5 Randomized crossover	Right FDI Right ADM	CRB: double-cone (110 mm) M1: figure-of-eight (90 mm)	Pre/Post 1–10 min, Post 10–20 min	Right CRB (FDI + ADM); ↓CBI ↔ MEP ↔ CBI, MEP
	6 HC	Right cervical root (control)	As above	As above		As above	As above	As above	Cervical roots (FDI, ADM): ↔ CBI, MEP
	6 HC	Left CRB	As above	As above		As above	As above	As above	Left CRB: ↓CBI (FDI, 10 min only) ↔ MEP (FDI, ADM)
	10 HC	Right CRB	cTBS iTBS	600 pulses 80 % Adj.AMT		As above	As above	As above	cTBS: ↓ CBI (FDI) ↔ MEP iTBS: ↔ CBI, MEP
Carrillo et al. (2013) [36]	16 HC	Right CRB	cTBS	600 pulses 80 % AMT	1	Right FDI	M1 and CRB: figure-of-eight (70 mm)	Pre/Post 0, 20, 40 min	HC: ↓ MEP, SICI
	13 PD				2 (On vs Off)				PD: ↔ MEP, SICI
Di Lorenzo et al. (2013) [40]	12 HC	Right lateral CRB	cTBS	600 pulses 80 % AMT	1	Right FDI	M1 and CRB: figure-of-eight (70 mm)	Pre/Post	HC: ↔ MEP, SICI, ICF, SLAI
	12 AD								AD: ↔ MEP, SICI, ICF; ↑ SLAI
	8 HC	Right lateral CRB	As above	As above	1	As above	As above	As above	↔ SAI_RC_
	8 HC	Right OC (control)	As above	As above	1	As above	As above	As above	↔ MEP, SICI, ICF, SLAI
Popa et al. (2013) [50]	14 HC	Right lateral CRB (Lobule VIII)	iTBS_CB_ → PAS_25_ cTBS_CB_ → PAS_25_ iTBS_CB_ → iTBS_M1_	600 pulses 80 % AMT	3, pseudo-randomized	Right APB Right ADM	M1 and CRB: figure-of-eight (70 mm)	Pre/Post 0, 5, 10, 15, 25, 45 min	↓ PAS_25_ (APB only) ↑ PAS_25_ (APB and ADM) ↔ iTBS_M1_
	9 HC	As above	cTBS_CB_ → iTBS_M1_	As above	1	As above	As above	As above	↔ iTBS_M1_
Hubsch et al. (2013) [49]	25 HC	Lobule VIII CRB	cTBS_CB_ → PAS_25_ iTBS_CB_ → PAS_25_ Sham_CB_ → PAS_25_	600 pulses 80 % AMT	3 randomized	Right APB Right ADM	M1 and CRB: figure-of-eight (70 mm)	Pre/Post 10, 15. 20, 25, 30 min	HC: cTBS: ↑ PAS_25_ iTBS: ↓ PAS_25_ All conditions : ↔ SICI, ICF, LICI, SAI, LAI
	21 WD								WD: cTBS_CB_: ↔ PAS_25_ iTBS_CB_: ↔ PAS_25_ All conditions : ↔ SICI, ICF, LICI, SAI, LAI
Kishore et al. (2014) [51]	16 PD with LIDs	CRB ipsi to affected side (Lobule VIII)	cTBS_CB_ → PAS_25_ Sham_CB_ → PAS_25_	600 pulses 80 % AMT	2 randomized	Contra. APB	M1 and CRB: figure-of-eight (70 mm)	Pre/Post 5, 15, 30 min	↑ PAS_25_ ↔ RMT, SICI, LICI, SAI, LAI
	16 HC	Right lateral CRB	As above	As above	2 randomized	Right APB	As above	As above	↔ PAS_25_
	7 PD with LIDs	CRB ipsi to affected side (Lobule VIII)	cTBS_CB_ → iTBS_M1_ Sham_CB_ → iTBS_M1_		2 randomized	Contra. APB	As above	As above	↔ iTBS_M1_
	20 PD with LIDs	Bilateral CRB (Lobule VIII)	cTBS_CB_ → iTBS_M1_ Sham_CB_ → iTBS_M1_	600 pulses 80 % AMT	10 (2 weeks) randomized groups	Right APB	As above	Pre/week 2, 4, 8 post	↑ PAS_25_ (week 2)
Bonnì et al. (2014) [58]	6 PCS	Damaged lateral CRB	iTBS	600 pulses 80 % AMT	10 (2 weeks)	Right FDI	M1 and CRB: figure-of-eight (70 mm)	Pre/Post	↓ CBI; ↑ ICF; ↔ SICI
Brusa et al. (2014) [59]	10 PSP	Left and right lateral CRB (2 min pause in between)	iTBS	600 pulses 80 % AMT	10 (2 weeks)	Right FDI	M1 and CRB: figure-of-eight (70 mm)	Pre/Post 2-week intervention *(no further information)*	↑CBI; ↔ MEP, SICI, ICF, SAI
Koch et al. (2014) [57]	10 CD	Bilateral CRB	cTBS	600 pulses 80 % AMT	10 (2 weeks)	Right FDI Right APB	M1 and CRB: figure-of-eight (70 mm)	Pre (Friday before the start of the 2-weeks treatment) Post (Monday after the end of the 2-weeks treatment)	Active (vs. sham): ↓ CBI; ↔ ICF, SICI, CSP ↑ PAS topographic specificity ↓ symptoms
	10 CD	Sham (coil angled 90°)	As above	600 pulses 40 % AMT	As above	As above	As above	As above	
Li Voti et al. (2014) [35]	12 HC	Right lateral CRB	cTBS	600 pulses 80 % AMT	1	Right FDI	M1 and CRB: figure-of-eight (70 mm)	Pre/Post 15, 30, 60 min	↓ MEP
Di Biasio et al. (2014) [33]	10 HC 15 PD OFF	Ipsi. Damaged CRB Sham (neck muscles)	cTBS	600 pulses 80 % AMT	2 randomized	Contra. FDI	M1 and CRB: figure-of-eight (90 mm)	Pre/Post 5, 25 min	HC and PD: ↓ MEP ↓ symptoms
Bologna et al. (2015) [38]	11 HC 16 ET	Right CRB Sham (neck muscles)	cTBS	600 pulses 80 % AMT	2 randomized	Right FDI	M1 and CRB: figure-of-eight	Pre/Post 5, 45 min	HC: ↓ MEP_RC_ ET: ↔ MEP_RC_ ↔ symptoms
Bologna et al. (2015) [37]	10 HC 13 RT	Ipsi. CRB to tremor hand Sham (neck muscles)	cTBS	600 pulses 80 % AMT	2 randomized	FDI (tremor hand)	M1 and CRB: figure-of-eight	Pre/Post 5, 45 min	HC and RT: ↓ MEP_RC_ ↔ symptoms
Harrington et al. (2015) [39]	13 HC	Right CRB	cTBS iTBS Sham TBS	600 pulses 80 % AMT (6 subjects) 90 % AMT (7 subjects)	3 randomized crossover	Right FDI	M1 and CRB: figure-of-eight	Pre/Post	cTBS: ↓ MEP

*AD* Alzheimer’s disease, *AMT* active motor threshold, *APB* abductor pollicis brevis, *CBI* cerebellar brain inhibition, *CBI*_*RC*_ cerebellar brain inhibition recruitment curve, *CRB* cerebellum, *Contra* contralateral, *CSP* cortical silent period, *ET* essential tremor, *FDI* first dorsal interosseous, *HC* healthy controls, *ICF* intracortical facilitation, *Isps* ipsilateral, *LICI* long interval intracortical inhibition, *M1* primary motor cortex, *MEP* motor evoked potential, *MEP*_*RC*_ motor evoked potential recruitment curve, *MSO* maximal stimulator output, *PAS* paired-associative stimulation, *PCS* posterior circulation stroke, *PD* Parkinson’s disease, *PSP* progressive supranuclear palsy, *SAI* short latency afferent inhibition, *SAI*_*RC*_ short latency afferent inhibition recruitment curve, *SICI* short interval intracortical inhibition, *SICF* short interval intracortical facilitation, *WD* writing dystonia

**Table 3:** Effect of cerebellar transcranial electrical stimulation on primary motor cortex excitability

Authors	Sample size	Electrode position	Polarity	Parameters	Sessions	Target muscle	Coil size	Timing of measurements	Findings
Galea et al. (2009) [41]	8 HC	Right CRB (25 cm^2^) Right buccinator muscle (25 cm^2^)	Anodal TDCS Cathodal TDCS Sham	2 mA 25 min	3 randomized crossover	FDI	M1: figure-of-eight (70 mm) CRB: double-cone (110 mm)	Pre/Post 0 min	Cathodal cDCS (vs sham): ↓ CBI ↔ MEP, SICI, ICF Anodal cDCS (vs sham): ↔ CBI, MEP, MT, SICI, ICF
	8 HC	As above	Anodal TDCS	As above	1	As above	As above	As above	↑ CBI_RC_
	6 HC	As above	Cathodal TDCS	1 mA 25 min 2 mA 25 min	2 randomized crossover	As above	As above	Pre/Post 0, 30, 50 min	1 mA: ↔ CBI, MEP_RC_ 2 mA: ↓ CBI, MEP_RC_
Hamada et al. (2012) [42]	12 HC	Right CRB (25 cm^2^) Right buccinator muscle (25 cm^2^)	Anodal TDCS-PAS25 Cathodal TDCS -PAS25 Sham TDCS -PAS25	2 mA 15 min	3 randomized crossover	APB	M1: figure-of-eight (70 mm)	Pre/Post 0, 30 min	Anodal and cathodal (vs sham): ↓ PAS25, ↔ SAI, MEP_RC_
	8 HC	As above	Anodal TDCS-PAS21.5 Sham TDCS-PAS21.5	As above	2 randomized crossover	As above	As above	As above	Anodal (vs sham): ↔ PAS21.5
Hamada et al. (2014) [44]	17 HC	Right lateral CRB (25 cm^2^) Right buccinator muscle (25 cm^2^)	Sham TDCS-PAS21.5 Sham TDCS-PAS25 Anodal TDCS-PAS21.5 Anodal TDCS-PAS25	2 mA 15 min	4 randomized crossover	APB	M1: figure-of-eight (70 mm)	Pre/Post 0, 15, 30 min	Anodal (vs sham): ↓ PAS25, ↔ PAS21.5
	10 HC	As above	Sham TDCS Anodal TDCS	2 mA 25 min	2 randomized crossover	As above	As above	Online (5 min after onset of stimulation)	Anodal (vs sham): ↓ MEP_RC_ (active AP) ↔ MEP_RC_ (active PA, rest PA and rest AP)
Sadnicka et al. (2014) [56]	10 WD	Right CRB (25 cm^2^) Right buccinator muscle (25 cm^2^)	Anodal TDCS-PAS25 Sham TDCS-PAS25	2 mA 15 min	2 randomized crossover	APB FDI ADM	M1: figure-of-eight (70 mm)	Pre/Post 0, 30 min	Anodal (vs sham): ↔ PAS25, CSP, MEP_RC_
Strigaro et al. (2014) [52]	8 HC	Right CRB (25 cm^2^) Right buccinator muscle (25 cm^2^)	Anodal TDCS-PASvar_360p_ Sham TDCS-PASvar_360p_	2 mA 30 min	2 randomized crossover	APB	M1: figure-of-eight (70 mm)	Pre/Post 0 min	Anodal (vs sham): ↑ PASvar_360p_
Doeltgen et al. (2015) [46]	14 HC	Right lateral CRB Right buccinator muscle	Anodal TDCS Sham	2 mA 20 min	2 randomized crossover	FDI	M1: figure-of-eight (70 mm) CRB: figure-of-eight	Pre/Post 0 min	Anodal (vs sham): ↓ CBI ↔ SAI
Naro et al. (2016) [45]	25 HC	Right CRB (25 cm^2^) Right buccinator muscle (25 cm^2^)	10 Hz TACS 50 Hz TACS 300 Hz TACS Sham TACS	2 mA 3000 cycles	4 randomized crossover	Right and left APB	M1: figure-of-eight CRB: double-cone	Pre/Post 0, 15, 30 min	50Hz TACS: ↓ CBI, ↑ MEP, ↔LICI 300Hz TACS: ↑ CBI, ↔MEP, LICI 10Hz TACS: ↔CBI, MEP, LICI

*AP* anterior-posterior, *APB* abductor pollicis brevis, *CBI* cerebellar brain inhibition, *CBI*_*RC*_ cerebellar brain inhibition recruitment curve, *CRB* cerebellum, *CSP* cortical silent period, *TACS* transcranial alternating current stimulation, *TDCS* cerebellar transcranial direct current stimulation, *FDI* first dorsal interosseous, *HC* healthy controls, *ICF* intracortical facilitation, *M1* primary motor cortex, *MEP* motor evoked potential, *MEP*_*RC*_ motor evoked potential recruitment curve, *PA* posterior-anterior, *PAS* paired-associative stimulation, *SAI* short latency afferent inhibition, *SICI* short interval intracortical inhibition, *WD* writing dystonia

**Table 4:** Effect of cerebellar-M1 paired-associative stimulation on primary motor cortex excitability

Authors	Sample size	Stimulation target(s)	Protocol	Parameters	Sessions	Target muscle	Coil size	Timing of measurements	Findings
Lu et al. (2012) [19]	13 HC	Right lateral CRB	CRB – M1 (PAS_2ms_)	CS: 90 % AMT 120 pairs 0.25 Hz	1	Left FDI	CRB: double-cone (110 mm) M1: figure-of-eight (90 mm)	Pre/Post 0, 30, 60 min	↑ MEP, ↓SICI CBI, ↔ICF
	6 HC	As above	CRB – M1 (PAS_6ms_)	As above	1	As above	As above	As above	↓ MEP, ↓SICI CBI, ↔ICF
	13 HC	As above	CRB – M1 (PAS_10ms_)	As above	1	As above	As above	As above	↓ MEP, ↓SICI CBI, ↔ICF
	9 HC	As above	CRB – M1 (PAS_control_ random 2, 6, 10 ms)	As above	1	As above	As above	As above	↔ MEP, SICI, CBI, ICF

*CBI* cerebellar brain inhibition, *CRB* cerebellum, *FDI* first dorsal interosseous, *HC* healthy controls, *ICF* intracortical facilitation, *M1* primary motor cortex, *MEP* motor evoked potential, *PAS* paired-associative stimulation, *SICI* short interval intracortical inhibition

**Table 5:** Effect of cerebellum modulation on M1 neurophysiology assessed with TMS in healthy individuals

Outcome measure	Plasticity protocol	Authors	Parameters	Findings
1. Corticospinal excitability				
Resting motor threshold	Anodal TDCS	Galea et al. (2009) [41]		↔
	Cathodal TDCS	Galea et al. (2009) [41]		↔
	1 Hz rTMS	Langguth et al. (2008) [47]		↔
	10 Hz rTMS	Langguth et al. (2008) [47]		↔
	cTBS	Di Lorenzo et al. (2013) [40]		↔
		Koch et al. (2008) [34]		↔
		Harrington et al. (2015) [39]		↔
	iTBS	Koch et al. (2008) [34]		↔
		Harrington et al. (2015) [39]		↔
MEP amplitude	Anodal TDCS	Galea et al. (2009) [41]	1 mV	↔
	Cathodal TDCS	Galea et al. (2009) [41]	1 mV	↔
	TACS	Naro et al. (2016) [45]	120 % RMT	↑ contralateral up to 15 min (50 Hz)
	1 Hz rTMS	Gerschlager et al. (2002) [29]	1–1.5 mV	↑ up to 30 min
		Oliveri et al. (2005) [30]	1 mV	↑ contralateral up to 15 min ↔ ipsilateral
		Fierro et al (2007) [31]	120 % RMT	↔ 5–10 min ↑ 15–20 min
		Popa et al. (2010) [32]	120 % RMT	↔
	cTBS	Koch et al. (2008) [34]	1 mV	↓ up to 15 min
		Popa et al. (2010) [32]	120 % RMT	↔
		Di Lorenzo et al. (2013) [40]	1 mV	↔
		Li Voti et al. (2014) [35]	1 mV	↓ up to 30 min
		Di Biasio et al. (2014) [33]	120 % RMT	↓
		Carrillo et al. (2013) [36]	0.5–1 mV	↓ up to 40 min
		Harrington et al. (2015) [39]	110 % RMT	↓ (rest) ↔ (active)
	iTBS	Koch et al. (2008) [34]	1 mV	↑ up to 15 min
		Popa et al. (2010) [32]	120 % RMT	↔
		Harrington et al. (2015) [39]	110 % RMT	↔ (rest and active)
	CB-M1 PAS	Lu et al. (2012) [19]	1 mV	↑ (PAS_2ms_) ↓ (PAS_6ms_, PAS_10ms_)
MEP recruitment curve	Anodal TDCS	Hamada et al. (2012) [42]	100, 120 and 140 % RMT	↔
		Hamada et al. (2014) [44]	100, 120, 140 and 160 % RMT	↔ PA rest (online) ↔ AP rest (online) ↔ PA active (online) ↓ AP active (online)
	Cathodal TDCS	Galea et al. (2009) [41]	100, 110, 120, 130 and 140 % RMT	↔ (1 and 2 mA)
		Hamada et al. (2012) [42]	100, 120 and 140 % RMT	↔
	cTBS	Bologna et al. (2015) [38]	100 to 150 % RMT	↓ 5 min, return to baseline 45 min
		Bologna et al. (2015b) [37]	100 to 140 % RMT	↓ up to 45 min
2. Cerebellum brain inhibition				
CBI	Anodal TDCS	Galea et al. (2009) [41]	ISI: 5 ms CS: 5 % below bsAMT, and 5, 10, 15, 20, 25 % below bsAMT TS 1 mV (adjusted post)	↑ CBI recruitment curve at 20–25 % below bsAMT
		Doeltgen et al. (2015) [46]	ISI: 5 ms CS: 100 % RMT (FDI) TS : 50 % MEP_MAX_	↓
	Cathodal TDCS	Galea et al. (2009) [41]	ISI: 3 and 5 ms CS: 5 % below bsAMT TS: 1 mV (adjusted post)	↓ (2 mA only, until 30 min post-TDCS) ↔ no CBI at 3 ms ISI
	TACS	Naro et al. (2016) [45]	ISI: 7 ms CS: 90 % AMT TS: 120 % RMT	↓ (50 Hz: up to 15 min post-TACS) ↑ (300 Hz: only 0 min post-TACS)
	1 Hz rTMS	Popa et al. (2010) [32]	ISI: 5 ms CS: 90 % adjusted-RMT TS: 120 % RMT	↓ (contralateral only, until 30 min post)
	cTBS	Popa et al. (2010) [32]	ISI: 5 ms CS: 90 % adjusted-RMT TS: 120 % RMT	↓
	iTBS	Popa et al. (2010) [32]	ISI: 5 ms CS: 90 % adjusted-RMT TS: 120 % RMT	↔
	CB-M1 PAS	Lu et al. (2012) [19]	ISI: 7 ms CS: 95 % AMT (inion) TS: 0.6–0.8 mV (FDI)	↓
**4.** Intracortical inhibition				
SICI	Anodal TDCS	Galea et al. (2009) [41]	ISI: 2 ms CS: 80 % RMT TS: 1 mV (adjusted post)	↔
	Cathodal TDCS	Galea et al. (2009) [41]	ISI: 2 ms CS: 80 % RMT TS: 1 mV (adjusted post)	↔
	1 Hz rTMS	Oliveri et al. (2005) [30]	ISI: 1 and 3 ms CS: 70 % RMT TS: 1 mV (adjusted post)	↔
		Fierro et al. (2007) [31]	ISI: 2 and 4 ms CS: 80 % RMT TS: 120 % RMT (adjusted post)	↔
		Langguth et al. (2008) [47]	ISI: 2, 3, 4 and 5 ms CS: 90 % AMT, TS: 1 mV	↑ (averaged ISIs)
	10 Hz rTMS	Langguth et al. (2008) [47]	ISI: 2, 3, 4 and 5 ms CS: 90 % AMT, TS: 1 mV	↔
	cTBS	Koch et al. (2008) [34]	ISI: 1, 2, 3, 4 and 5 ms CS: 80 % AMT, TS: 1 mV	↓ (3 ms, contralateral only)
		Carrillo et al. (2013) [36]	ISI: 1, 2, 3, 4 and 5 ms CS: 80 % AMT TS: 1 mV	↓ (2 and 3 ms, 0–20 min)
		Di Lorenzo et al. (2013) [40]	ISI: 1, 2, 3, 4 and 5 ms CS: 80 % AMT TS: 1 mV	↔
		Hubsch et al. (2013) [49]	SI: 2.5 ms CS: 70 % RMT TS: 130 % RMT (adjusted post)	↔
	iTBS	Koch et al. (2008) [34]	ISI: 1, 2, 3, 4 and 5 ms CS: 80 % AMT TS: 1 mV	↔
	CB-M1 PAS	Lu et al. (2012) [19]	ISI: 2 ms CS: 70 to 90 % AMT (50 % inh.)	↓ (all PAS ISIs)
LICI	TACS	Naro et al. (2016) [45]	ISI: 50 ms CS: 120 % RMT TS: 120 % RMT	↔
	cTBS	Koch et al. (2008) [34]	ISI: 100 and 150 ms CS: 120 % RMT TS: 1 mV	↑ (100 ms)
		Hubsch et al. (2013) [49]	SI: 100 ms CS: 120 % RMT TS: 130 % RMT (adjusted post)	↔
	iTBS	Koch et al. (2008) [34]	ISI: 100 and 150 ms CS: 120 % RMT TS: 1 mV	↓ (100 ms)
CSP	1 Hz rTMS	Oliveri et al. (2005) [30]	30 % maximal force TS: 1 mV	↔
	cTBS	Harrington et al. (2015) [39]	20 Newton force TS:110 % RMT	↔
	iTBS	Harrington et al. (2015) [39]	20 Newton force TS:110 % RMT	↔
**5.** Intracortical facilitation				
ICF	Anodal TDCS	Galea et al. (2009) [41]	ISI: 10 ms CS: 80 % RMT TS: 1 mV (adjusted post)	↔
	Cathodal TDCS	Galea et al. (2009) [41]	ISI: 10 ms CS: 80 % RMT TS: 1 mV (adjusted post)	↔
	1 Hz rTMS	Oliveri et al. (2005) [30]	ISI: 7, 10 and 15 ms CS: 70 % RMT TS: 1 mV (adjusted post)	↑ (15 ms)
		Fierro et al (2007) [31]	ISI: 7, 10 and 15 ms CS: 80 % RMT TS: 120 % RMT	↓ (10 ms)
		Langguth et al. (2008) [47]	ISI: 7, 8, 10, 15 and 20 ms CS: 90 % AMT TS: 1 mV	↑ (15 and 20 ms)
	10 Hz rTMS	Langguth et al. (2008) [47]	ISI: 7, 8, 10, 15 and 20 ms CS: 90 % AMT TS: 1 mV	↔
	iTBS	Koch et al. (2008) [34]	ISI: 7, 10 and 15 ms CS: 80 % AMT TS: 1 mV	↓ (15 ms)
	cTBS	Koch et al. (2008) [34]	ISI: 7, 10 and 15 ms CS: 80 % AMT TS: 1 mV	↔
		Carrillo et al. (2013) [36]	ISI: 7, 10 and 15 ms CS: 80 % AMT TS: 1 mV	↔
		Di Lorenzo et al. (2013) [40]	ISI: 7, 10 and 15 ms CS: 80 % AMT TS: 1 mV	↔
		Hubsch et al. (2013) [48]	SI: 15 ms CS: 70 % RMT TS: 130 % RMT (adjusted post)	↔
	CB-M1 PAS	Lu et al. (2012) [19]	ISI: 10 ms CS: 70 to 95 % AMT	↔
SICF	cTBS	Koch et al. (2008) [34]	ISI: 1.0, 1.3, 2.1, 2.5, 3.3, 4.1 ms CS: 90 % RMT TS: 130 % RMT	↔
	iTBS	Koch et al. (2008) [34]	ISI: 1.0, 1.3, 2.1, 2.5, 3.3, 4.1 ms CS: 90 % RMT TS: 130 % RMT	↔
**6.** Afferent inhibition				
SAI	Anodal TDCS	Hamada et al. (2012) [42]	ISI: 15, 20 and 25 ms TS: 1 mV	↔
		Doeltgen et al. (2015) [46]	ISI: 25 and 30 ms TS : 50 % MEP_MAX_	↔
	Cathodal TDCS	Hamada et al. (2012) [42]	ISI: 15, 20 and 25 ms TS: 1 mV	↔
	cTBS	Di Lorenzo et al. (2013) [40]	ISI: N20 – 4 ms to N20 + 8 ms TS: 1 mV	↔
		Hubsch et al. (2013) [49]	ISI: 20 ms TS: 130 % RMT (adjusted post) CS: 130 % sensory threshold	↔
SAI recruitment curve	cTBS	Di Lorenzo et al. (2013) [40]	ISI: N20 – 4 ms to N20 + 8 ms TS: 1 mV CS: 100, 200 and 300 % sensory threshold	↔
LAI	cTBS	Hubsch et al. (2013) [49]	ISI: 200 ms TS: 130 % RMT (adjusted post) CS: 130 % sensory threshold	↔
**7.** Motor cortex plasticity				
PAS	Anodal TDCS	Hamada et al. (2012) [42]	ISI: 21.5 and 25 ms	↓ PAS_25_ ↔ PAS_21.5_
		Hamada et al. (2014) [44]	ISI: 21.5 and 25 ms	↓ PAS_25_ ↔ PAS_21.5_
		Strigaro et al. (2014) [52]	ISI: 21.5, 25 ms and variable	↑ PAS_var_
	Cathodal TDCS	Hamada et al. (2012) [42]	ISI: 21.5 and 25 ms	↓ PAS_25_
	cTBS	Popa et al. (2013) [50]	ISI: 25 ms	↑ PAS_25_ (post 25–60 min)
		Hubsch et al. (2013) [49]	ISI: 25 ms	↑ PAS_25_
		Kishore et al. (2014) [51]	ISI: 25 ms	↔ PAS_25_
	iTBS	Popa et al. (2013) [50]	ISI: 25 ms	↓ PAS_25_ (post 5–15 min)
		Hubsch et al. (2013) [49]	ISI: 25 ms	↓PAS_25_
cTBS	iTBS	Popa et al. (2013) [50]	80 % AMT, 600 pulses Contra. M1	↔
iTBS	iTBS	Popa et al. (2013) [50]	80 % AMT, 600 pulses Contra. M1	↔

*AMT* active motor threshold, *CBI* cerebellar brain inhibition, *CS* conditioning stimulus, *Contra* contralateral, *CSP* cortical silent period, *HC* healthy controls, *ICF* intracortical facilitation, *Ipsi*. ipsilateral, *ISI* inter-stimulus interval, *LAI* long latency afferent inhibition, *LICI* long interval intracortical inhibition, *MEP* motor evoked potential, *PAS* paired-associative stimulation, *SAI* short latency afferent inhibition, *SICI* short interval intracortical inhibition, *SICF* short interval intracortical facilitation, *TS* test stimulus

#### Effect of cerebellar stimulation on corticospinal excitability

None of the studies reports an effect of cerebellar “plasticity” paradigms on RMT. In contrast, MEPs evoked by a standard suprathreshold TMS pulse (usually set to produce a baseline average MEP of 1 mV peak-to-peak amplitude) may change. The effect is seen in M1 contralateral to the side of cerebellar stimulation and hence is appropriate for a cerebellar-induced effect.

However, the findings are variable and sometimes contradictory. Thus, cerebellar 1Hz rTMS (rTMS_CB_) has been investigated in four studies. Gerschlager et al. [29] were the first to assess the effect of rTMS_CB_ on M1 MEP amplitude and found a significant increase that lasted up to 30 min after stimulation. This was substantiated by two studies [30, 31], although a more recent study found no significant change [32]. Cerebellar cTBS (cTBS_CB_), which like 1 Hz rTMS is usually claimed to have an inhibitory effect on M1 excitability, appears to have an opposite effect on cerebellum: cTBS_CB_ reduced MEP amplitudes in 7 studies (and in 2 of them it also reduced the slope of the MEP recruitment curve) [33–39], but had no effect in two others [32, 40]. Cerebellar iTBS (iTBS_CB_) was reported to increase MEPs in one study [34] but there was no effect in two studies [32, 39].

Cerebellar TDCS (TDCS_CB_) has never been reported to have any effect on MEP amplitude or MEP_RC_ following either anodal or cathodal stimulation [41–43]. In contrast to the usual “offline” study (i.e. where MEPs are evaluated before and after TDCS), Hamada et al. [44] noted an effect on MEPs if they were assessed *during* TDCS_CB_. However, the effect could only be observed if MEPs were evoked by low intensity stimuli in actively contracting muscle using an antero-posterior induced current in M1. It is therefore possible that the effect of TDCS_CB_ on M1 excitability may be masked when MEPs are assessed with a suprathreshold stimulus applied using the standard posterior-anterior current direction.

Two further sets of observations have been reported but not yet replicated. In one of them 50 Hz TACS increased MEP amplitudes [45]. The other used a novel cerebellar-M1 paired-associative protocol in an attempt to engage STDP mechanisms [19]. One hundred and twenty pairs of cerebellum/M1 TMS pulses applied with an interstimulus interval of 2 ms increased MEPs whereas ISIs of 6 and 10 ms decreased MEPs.

#### Effect of cerebellar NIBS on CBI

Only 5 articles have reported effects on CBI. Most of them report reductions in the effectiveness of CBI: this occurs after 1 Hz rTMS_CB_ or cTBS_CB_ [32]; after TACS_CB_ [45]; after cathodal TDCS_CB_ [41]; and after cerebellar-M1 PAS at any ISI [19]. Anodal TDCS_CB_ has been tested by two groups who obtained opposite answers: Galea et al. found an increase in CBI [41] whereas Doeltgen and colleagues described a reduction [46]. However, the parameters for assessing CBI differed in the two studies.

#### Effect of cerebellar NIBS on intracortical interactions in M1

In addition to effects on MEP excitability, there are a number of reports in which local inhibitory and facilitatory interactions within M1 have been studied. However, the evidence for definitive effects is sparse, and more studies are needed.

#### Short interval intracortical inhibition (SICI)

No effects were observed after anodal and cathodal TDCS_CB_, 10 Hz rTMS_CB_ and iTBS_CB_ [34, 41, 47]. There is one report of increased SICI after 1Hz rTMS_CB_ [47] but two others reported no change [30, 31]. Two studies reported a reduction of SICI after cTBS_CB_, [36, 48] but there was no effect in two other studies [40, 49]. There is one report that cerebellar-M1 PAS reduced SICI at all ISIs tested [19].

#### Intracortical facilitation (ICF)

As with SICI, only a few studies provide evidence that cerebellar “plasticity” protocols have an effect on ICF. No effects were observed after cTBS_CB_ [34, 36, 40, 49], TDCS_CB_ [41], 10 Hz rTMS_CB_ [47] and CB-M1 PAS [19]. Reduced ICF was reported following iTBS_CB_ [34]. Two studies reported that 1 Hz rTMS_CB_ increased ICF levels [30, 47] and a third [31] observed a trend towards an increase of ICF using a 15 ms ISI and a significant decrease at an ISI of 10 ms.

#### Other protocols

There is very little data available for other protocols. LICI was reported to be unchanged by TACS_CB_ [45], increased by cTBS_CB_ [34, 49], and decreased by iTBS_CB_ [34]. No change in the CSP was seen after 1 Hz rTMS_CB_ [30], and both iTBS and cTBS [39]. SICF was unaffected by continuous or intermittent TBS_CB_ [34], whilst no effects were observed after anodal TDCS_CB_ [42, 46], cathodal TDCS_CB_ [42], or cTBS_CB_ [40, 49] for SAI. LAI was unchanged following cTBS_CB_ [49].

#### Cerebellar interactions with M1 plasticity

Most studies have focused on the impact of cerebellar modulation on motor cortex paired-associative stimulation (PAS). PAS entails pairing an afferent sensory input (usually median nerve stimulation) with a suprathreshold TMS pulse applied to motor cortex after a short interval. Adjusting this interstimulus interval varies the effect of the protocol in a way that mirrors the effect seen with animal models of spike-timing dependent plasticity. It is generally agreed that ISIs of 21.5 – 25 ms are facilitatory. In the reviewed articles, 5 out of 6 studies report significant interactions, and suggest that the effects are mediated by an effect of cerebellar activity on transmission of sensory input from median nerve to M1.

Popa et al. [50] found that cerebellar cTBS increased the amplitude, duration and spatial extent of the response to PAS25 (i.e. PAS with a 25 ms interval between median nerve stimulation and M1 TMS), whereas cerebellar iTBS blocked the effect of PAS25. Similar results were reported by Hubsch et al. [49], while no effect of cTBS_CB_ on PAS25 was found by Kishore et al. [51]. In contrast, neither form of cerebellar TBS affected the response to motor cortex iTBS, consistent with the cerebellum being involved in the afferent arm of the PAS protocol.

Rather than examining the *offline* effects of cerebellar interventions, a series of studies reported the effects of *online* TDCS_CB_. Hamada et al. [42] found that both anodal and cathodal TDCS_CB_ blocked the effect of PAS25. However, they found that anodal TDCS_CB_ had no effect on the response to PAS21.5. They argued that this was compatible with the idea that PAS21.5 and PAS25 have different mechanisms. One possibility was that PAS25 utilised an afferent pathway from median nerve to M1 that traversed cerebellar pathways, whereas PAS21.5 represented an interaction with more direct lemniscal inputs. Results compatible with this hypothesis were reported by Strigaro et al. [52].

### Primary motor cortex changes following cerebellar stimulation in clinical populations

The current systematic review identified 12 studies involving six different neurological disorders. Interestingly, 11 out of the 12 studies investigated the effect of intermittent or continuous TBS_CB_. One study assessed the effect of TDCS_CB_, whereas CB-M1 PAS and low- or high-frequency rTMS have not been investigated. Main findings for each clinical population will be briefly described below. See Table 6 for a complete description of results for each M1 outcome measure.

**Table 6:** Effect of cerebellum modulation on M1 neurophysiology assessed with TMS in clinical populations

Outcome measure	Plasticity protocol	Authors	Population	Parameters	Findings
1. Corticospinal excitability					
Resting motor threshold	cTBS	Di Lorenzo et al. (2013) [40]	AD		↔ *↔ (HC)*
	cTBS	Kishore et al. (2014) [51]	PD with LIDs		↔
MEP amplitude	cTBS	Di Lorenzo et al. (2013) [40]	AD	1 mV	↔ *↔ (HC)*
		Di Biasio et al. (2015) [33]	PD	120 % RMT	↓ Off medication *↓ (HC)*
	iTBS	Carrillo et al. (2013) [36]	PD	0.5–1 mV	↔ On or Off medication *↓ (HC)*
		Brusa et al. (2014) [59]	PSP	1 mV	↔
MEP recruitment curve	cTBS	Bologna et al. (2015) [38]	ET	100 to 150 % RMT	↔ *↓ (HC)*
		Bologna et al. (2015b) [37]	RT (PD)	100 to 140 % RMT	↓ up to 45 min *↓ (HC)*
		Sadnicka et al. (2014) [56]	WD	100 to 140 % RMT	↔
2. Cerebellum brain inhibition					
CBI	cTBS	Koch et al. (2014) [57]	CD	ISI: 3, 5, 10 ms CS: 90 % RMT (ipsi. M1) TS: 1 mV	↓ ISI 10 ms
	iTBS	Bonnì et al. (2014) [58]	PCS	ISI: 3, 5, 10 ms CS: 90 % RMT (contra. M1) TS: 0.5–1 mV	↓ all ISIs
		Brusa et al. (2014) [59]	PSP	ISI: 3, 5, 10 ms CS: 90 % RMT (ipsi. M1) TS: 1 mV	↑ all ISIs
3. Intracortical inhibition					
SICI	cTBS	Koch et al. (2009) [48]	PD	ISI: 1, 2, 3, 4 and 5 ms CS: 80 % AMT TS: 1 mV	↓
		Carrillo et al. (2013) [36]	PD	ISI: 1, 2, 3, 4 and 5 ms CS: 80 % AMT TS: 1 mV	↔ *↓ (HC)*
		Di Lorenzo et al. (2013) [40]	AD	ISI: 1, 2, 3, 4 and 5 ms CS: 80 % AMT TS: 1 mV	↔ *↔ (HC)*
		Hubsch et al. (2013) [49]	WD	SI: 2.5 ms CS: 70 % RMT TS: 130 % RMT (adj. post)	↔ *↔ (HC)*
		Koch et al. (2014) [57]	CD	ISI: 1, 2, 3, 4 and 5 ms CS: 80 % AMT TS: 1 mV	↔
		Kishore et al. (2014) [51]	PD with LIDs	ISI: 2.5 ms CS: 70 % RMT TS: 1 mV	↔
	iTBS	Bonnì et al. (2014) [58]	PCS	ISI: 1, 2, 3, 4 and 5 ms CS: 80 % AMT TS: 1 mV	↔
		Brusa et al. (2014) [59]	PSP	ISI: 1, 2, 3, 4 and 5 ms CS: 80 % AMT TS: 1 mV	↔
LICI	cTBS	Koch et al. (2009) [48]	PD with LID	ISI: 100 and 150 ms CS: 120 % RMT TS: 1 mV	↑ 100 ms
		Hubsch et al. (2013) [49]	WD	SI: 100 ms CS: 120 % RMT TS: 130 % RMT (adj. post)	↔ *↔ (HC)*
		Kishore et al. (2014) [51]	PD with LIDs	ISI: 100 ms CS: 110 % RMT TS: 1 mV	↔
CSP	Anodal TDCS	Sadnicka et al. (2014) [56]	WD	20 % maximal force APB TS: 120 % RMT	↔
	cTBS	Koch et al. (2014) [57]	CD	50 % maximal force TS: 130 % RMT	↔
4. Intracortical facilitation					
	cTBS	Koch et al. (2009) [48]	PD	ISI: 7, 10 and 15 ms CS: 80 % AMT TS: 1 mV	↔
		Carrillo et al. (2013) [36]	PD	ISI: 7, 10 and 15 ms CS: 80 % AMT TS: 1 mV	↔ *↔ (HC)*
		Di Lorenzo et al. (2013) [40]	AD	ISI: 7, 10 and 15 ms CS: 80 % AMT TS: 1 mV	↔ *↔ (HC)*
		Hubsch et al. (2013) [49]	WD	SI: 15 ms CS: 70 % RMT TS: 130 % RMT (adj. post)	↔ *↔ (HC)*
		Koch et al. (2014) [57]	CD	ISI: 7, 10 and 15 ms CS: 80 % AMT TS: 1 mV	↔
	iTBS	Bonnì et al. (2014) [58]	PCS	ISI: 7, 10 and 15 ms CS: 80 % AMT TS: 1 mV	↑ 15 ms
		Brusa et al. (2013) [59]	PSP	ISI: 7, 10 and 15 ms CS: 80 % AMT TS: 1 mV	↔
5. Afferent inhibition					
SAI	iTBS	Brusa et al. (2014) [59]	PSP	ISI: 16, 20, 24 and 28 ms TS: 1 mV	↔
	cTBS	Di Lorenzo et al. (2013) [40]	AD	ISI: N20–4 ms to N20 + 8 ms TS: 1 mV	↑ *↔ (HC)*
		Hubsch et al. (2013) [49]	WD	ISI: 20 ms TS: 130 % RMT (adj.post) CS: 130 % sensory threshold	↔ *↔ (HC)*
		Kishore et al. (2014) [51]	PD with LIDs	ISI: 20 ms TS: 1 mV	↔
LAI	cTBS	Hubsch et al. (2013) [49]	WD	ISI: 200 ms TS: 130 % RMT (adj. post) CS: 130 % sensory threshold	↔ *↔ (HC)*
6. Motor cortex plasticity					
PAS	Anodal TDCS	Sadnicka et al. (2014) [56]	WD	ISI: 25 ms	↔
	cTBS	Hubsch et al. (2013) [49]	WD	ISI: 25 ms	↔
		Koch et al. (2014) [57]	CD	ISI: 25 ms	↑ (topographic specificity)
		Kishore et al. (2014) [51]	PD with LIDs	ISI: 25 ms	↑ *↔ (HC)*
	iTBS	Hubsch et al (2013) [49]	WD	ISI: 25 ms	↔
iTBS	cTBS	Kishore et al. (2014) [51]	PD with LIDs	ISI: 25 ms	↔

*AD* Alzheimer’s disease, *AMT* active motor threshold, *CBI* cerebellar brain inhibition, *CS* conditioning stimulus, *Contra* contralateral, *CSP* cortical silent period, *ET* essential tremor, *HC* healthy controls, *ICF* intracortical facilitation, *Ipsi*. ipsilateral, *ISI* inter-stimulus interval, *LAI* long latency afferent inhibition, *LICI* long interval intracortical inhibition, *LIDs* levodopa-induced dyskinesias, *MEP* motor evoked potential, *PAS* paired-associative stimulation, *PCS* posterior circulation stroke, *PD* Parkinson’s disease, *PSP* progressive supranuclear palsy, *RT* resting tremors, *SAI* short latency afferent inhibition, *SICI* short interval intracortical inhibition, *TS* test stimulus, *WD* writing dystonia

#### Parkinson’s disease

Although Parkinson’s disease (PD) is primarily associated with degeneration of the dopaminergic nigrostriatal pathways, recent studies have suggested that cerebellar circuits could be a potential therapeutic target [53]. For example, there is evidence for the presence of cerebellar hyperactivity in PD patients, which could either be compensating or contributing to motor deficits [54]. If the latter is true, then reducing cerebellar activity could restore normal interactions between M1 and the cerebellum [36], and have a positive impact on symptoms. The effect of a single (5 studies) and multiple (1 study) session(s) of cTBS_CB_ were assessed in this population.

In detail, in PD patients displaying levodopa-induced dyskinesia (LID), results from Koch et al. [48] show that a single session of cTBS_CB_ can modify M1 intracortical circuits (decreased SICI and increased LICI). While Kishore and colleagues [51] did not replicate this result, they show that both a single session as well as 10 sessions of cTBS_CB_ increase the effect of PAS25 applied over M1 and reduced symptoms of dyskinesia. In PD patients off dopaminergic therapy, decreased M1 cortical excitability was induced by a single session of of cTBS_CB_ in two studies [33, 55], although only one of those was paralleled by functional changes, i.e. improvements in somatosensory temporal discrimination in PD patients off therapy [33]. In contrast, in PD patients displaying probable abnormal DTC pathway activity at baseline (reduced CBI levels), cTBS_CB_ did not modulate M1 cortical excitability and inhibition [36]. CBI levels were not reassessed following theta burst stimulation. Although current evidence remains limited, these studies suggest that the cerebellum may be involved in specific aspects of the pathophysiology of PD, such as levodopa-induced dyskinesias and altered sensory discrimination.

#### Dystonia

Dystonia is a movement disorder characterised by excessive involuntary muscle contraction. In the context of the present review, focal dystonia, i.e. cervical and writer’s dystonia, has been studied (three studies in total). In writer’s dystonia patients, Hubsch et al. [49] assessed the impact of cTBS_CB_, iTBS_CB_ and sham TBS_CB_ on subsequent PAS applied to M1. As opposed to healthy individuals, patients did not display modulations of PAS. Similar findings were observed in a separate study in cervical dystonia that used anodal TDCS_CB_ and showed no impact on subsequent PAS applied to M1 [56]. These two studies suggest that loss of cerebellar control over sensorimotor plasticity could underlie alterations of specific motor programs involved in writing. In a sham controlled trial involving 2-weeks of cTBS_CB_ in twenty patients with cervical dystonia, “active” stimulation resulted in reduced CBI levels, as well as increased sensorimotor topographic-specific plasticity (PAS) and clinical improvements [57]. However, no changes were observed regarding levels of M1 intracortical inhibition (SICI, CSP) and facilitation (ICF). Results from this study suggest that targeting the cerebellum could help restore normal M1-CB pathways and reduce symptoms of cervical dystonia.

#### Posterior circulation stroke

Cerebellar ataxia is a common impairment after posterior circulation stroke (PCS). One study [58] found that 10 sessions of iTBS_CB_ applied over a 2-week period increased the excitability of M1 facilitatory circuits that were found to be defective at baseline (elevated ICF prior to iTBS_CB_), while SICI levels remained unchanged. As iTBS_CB_ also reduced CBI in patients, the authors hypothesized that changes in M1 facilitatory circuits could have been mediated by a reduction in cerebellar tonic inhibition over M1. However, generalization of the results from this study is limited by the lack of a sham condition or control group.

#### Progressive supranuclear palsy

Progressive supranuclear palsy (PSP) is a parkinsonian syndrome characterised by symptoms such as postural instability. Cerebellar dentate nucleus dysfunction is thought to be involved. A single study assessed the effect of 10 sessions of iTBS_CB_ applied over a 2-week period in 10 patients with PSP [59]. No impact was found on motor inhibitory (SICI) and facilitatory circuits (ICF) or in sensorimotor inhibition. Although iTBS_CB_ did not modulate CBI in the single study performed with healthy controls (see [32]), it successfully increased the abnormally low levels of CBI observed at baseline in these patients [59]. Importantly, this was paralleled by clinical improvements. Although it remains to be replicated in a sham controlled experiment, this study suggests that applying iTBS to the cerebellum can potentially modulate the cerebellar-cortical pathway and alleviate symptoms in this clinical population.

#### Essential tremor

Essential tremor (ET) is a common movement disorder characterized by a combination of postural and kinetic tremors. The pathophysiology of the disorder is thought to involve the cerebello-thalamo-cortical loops and probable cerebellar hyperactivity [60]. Bologna and colleagues [38] studied the effect of a single session of active versus sham cTBS_CB_ in 15 patients with ET compared with 10 healthy individuals. As opposed to control subjects, cTBS_CB_ did not change M1 excitability in ET patients. There was no effect on clinical tremor. This study points towards the presence of probable abnormal cerebello-thalamo-cortical connectivity or abnormal cerebellar plasticity or function in ET. However, as CBI was not assessed in these patients, this study does not allow to distinguish the involvement of either probable cerebellar hyperexcitability or abnormal connectivity with motor cortex.

#### Alzheimer’s disease

Alzheimer’s disease (AD) is characterized by progressive neuronal degeneration that eventually affects cortical and subcortical regions, such as the cerebellum and primary motor and sensory cortices. Di Lorenzo et al. [40] studied the effect of a single session of cTBS_CB_ in 12 patients with AD and 12 healthy individuals. They showed that cTBS_CB_ could restore the initially reduced level of SAI to healthy controls levels [40], implying that the cerebellum may have direct influence on cholinergic and GABAergic dysfunctions in AD.

## Conclusions

In this systematic review of the literature, results from 27 studies which assessed the impact of cerebellar non-invasive “plasticity” protocols on TMS measures of M1 activity were reviewed. The main conclusion is that apart from CBI, produced by high intensity single pulse stimulation, all other protocols lack consistency and require further study in larger numbers of individuals. This is not surprising since most of the reviewed studies were underpowered with an average of only 11 subjects for the main experiments (ranging from 6 to 25).

Despite this rather negative conclusion, there are two relatively consistent effects. One of them is reduced CBI following cerebellar rTMS or TDCS/TACS. Facilitation of CBI was seen in one study after anodal TDCS, but this was not replicated in another study. Inhibition of CBI was found regardless of the inhibitory or excitatory impact that the same protocols might have on M1. Why this is the case is unknown. It could be that the mechanisms of cerebellar after-effects differ from those in cortex, perhaps because they target different neuronal types and pathways: alternatively it could simply reflect the well-known variability of rTMS/TDCS effects and be a chance phenomenon.

A second repeatable consequence is an effect on spike-timing dependent plasticity assessed in M1, i.e. PAS. Cerebellar stimulation affected median nerve PAS when it was evoked with an ISI of 25 ms (PAS25) but not with an interval of 21.5 ms (PAS21.5). Hamada et al. [42] suggested that cerebellar NIBS might act by altering sensory signals reaching M1 via the cerebellum (PAS25), while more direct afferent signals may be unaltered by cerebellar stimulation (PAS21.5). A recent study conducted in patients with cerebellar degeneration also points towards the implication of the cerebellum in PAS25, without affecting PAS21.5 [61]. Of note, cerebellar NIBS did not modify M1 response to TBS which would be consistent with an effect targeting the afferent input pathway of PAS.

Changes in M1 excitability (MEP amplitude) and paired pulse measures of M1 inhibition and facilitation are inconsistent. The studies on patients are too sparse to make any definitive conclusions.

### Current limitations and future directions

The main limitation in all these studies is that as yet we have no information about what is stimulated and where it is. For M1, for example, we have direct evidence in primates and in humans from pyramidal tract recordings in spinal cord that TMS activates M1 output, and that the after-effects of rTMS/TDCS protocols can modulate the response of this output to TMS. Brain imaging studies show lasting effects on metabolism and on levels of neurotransmitters, but there is no comparable data for the cerebellum. The best indirect evidence for changes in cerebellar output comes from CBI, which is thought to activate Purkinje cells of the cerebellum because of its high intensity and latency of effects. However, as noted in the Introduction, even this can be questioned. “Plasticity” protocols for the cerebellum employ stimulus intensities smaller than used for CBI and therefore evidence of their action is indirect, and probably involve synaptic inputs projecting to the Purkinje cells. Some authors have hypothesized that the effects of those protocols may be mediated by the activation of low-threshold interneurons leading to pre and post synaptic interactions at the Purkinje cell synapse which in turn modulate the output of the dentate nucleus and the DTC pathway resulting in changes in M1 excitability [34]. However, this remain highly hypothetical and further studies should investigate the effect of modifying “plasticity” paradigms to account for the anatomical characteristics of the cerebellum, e.g. use of higher stimulation intensities and longer durations or “spaced” repeated sessions for TBS.

This review also highlights a lack of consistency in parameters used for stimulation across studies. For example, some studies have used a constant stimulation intensity (40 % MSO) for repetitive TMS, while other studies based the intensity on resting or active thresholds measured over M1 or on an adjusted RMT that takes into account the distance between the coil and the cerebellum. Additionally, there is high variability in intensity (e.g. percentage of brainstem threshold, of adjusted motor threshold, of resting motor threshold and of active motor threshold) and intervals (e.g. 3 to 7 ms) used to assess CBI. This may explain some of the discrepancy among studies. For example, Galea and collaborators [41] showed that CBI is modified following anodal TDCS only at intensities of 20–25 % of brainstem threshold. These inconsistencies and the lack of a systematic assessment of those parameters may contribute to the observed lack of clear pattern of changes for M1 excitability and may significantly influence the ability to effectively modulate the lateral cerebellum. Further studies should also investigate if the same rules of M1 NIBS apply to the cerebellum, such as bidirectional changes and the effect of prior muscle contraction on the ability to induce plastic changes.

Brain imaging could in the future help to test our ideas about how these methods influence activity in cerebellum and its projections, and assess for optimal stimulation parameters. More detailed animal models of direct recordings of cell activity could also help confirm the physiological mechanisms underlying cerebellar modulation and CBI. Studies which model the distribution of electric field produced by stimulation can also give some indication of likely mechanisms of action. However, such studies are complex because of the need to integrate field calculations with individual neural geometry, and as such they only remain “models” until tested adequately with experimental methods.

Although the above-mentioned limitations currently restrict the clinical application of cerebellar modulation, results from the 12 studies involving clinical populations showed that as for healthy controls, CBI can be reliably targeted by cerebellar NIBS. Findings from clinical studies also suggest that cerebellar modulation can provide valuable information on the integrity of the DTC pathway and sensorimotor plasticity mechanisms in M1, especially in the case of Parkinson’s disease and cervical dystonia. Although this suggests that cerebellar modulation holds promise in rehabilitation of the DTC pathway and cerebellar-M1 abnormal activity, clinical studies using cerebellar NIBS remain limited. For instance, several NIBS methods studied in healthy individuals, such as low-frequency rTMS, CB-M1 PAS and TACS, lack comparative studies in clinical populations. In addition, very few studies included a control group or a sham condition, and as for healthy populations, there is a lack of consistency in parameters used for stimulation.

## Abbreviations

AD: Alzheimer’s disease
CB: Cerebellar
CBI: Cerebellar brain inhibition
CSP: Cortical silent period
cTBS: Continuous theta burst stimulation
DTC: Dentate-thalamo-cortical
EPSP: Excitatory post-synaptic potentials
ET: Essential tremor
ICF: Intracortical facilitation
iTBS: Intermittent theta burst stimulation
LAI: Long latency afferent inhibition
LICI: Long interval intracortical inhibition
M1: Primary motor cortex
MEP: Motor evoked potential
MEP_RC_: Motor evoked potential recruitment curve
PAS: Paired-associative stimulation
PCS: Posterior circulation stroke
PD: Parkinson’s disease
PSP: Progressive supranuclear palsy
RMT: Resting motor threshold
rTMS: Repetitive transcranial magnetic stimulation
SAI: Short latency afferent inhibition
SICF: Short interval intracortical facilitation
SICI: Short interval intracortical inhibition
STDP: Spike-timing dependent plasticity
TACS: Transcranial alternating current stimulation
TBS: Theta burst stimulation
TDCS: Transcranial direct current stimulation
TMS: Transcranial magnetic stimulation
